# Field-driven Domain Wall Motion in Ferromagnetic Nanowires with Bulk Dzyaloshinskii-Moriya Interaction

**DOI:** 10.1038/srep25122

**Published:** 2016-04-27

**Authors:** Fengjun Zhuo, Z. Z. Sun

**Affiliations:** 1 College of Physics, Optoelectronics and Energy & Jiangsu Key Laboratory of Thin Films, Soochow University, Suzhou, Jiangsu 215006, China; 2 Jiangsu Key Laboratory for Carbon-Based Functional Materials & Devices, Institute of Functional Nano & Soft Materials (FUNSOM), Soochow University, Suzhou, Jiangsu 215123, China

## Abstract

Field-driven domain wall (DW) motion in ferromagnetic nanowires with easy- and hard-axis anisotropies was studied theoretically and numerically in the presence of the bulk Dzyaloshinskii-Moriya interaction (DMI) based on the Landau-Lifshitz-Gilbert equation. We propose a new trial function and offer an exact solution for DW motion along a uniaxial nanowire driven by an external magnetic field. A new strategy was suggested to speed up DW motion in a uniaxial magnetic nanowire with large DMI parameters. In the presence of hard-axis anisotropy, we find that the breakdown field and velocity of DW motion was strongly affected by the strength and sign of the DMI parameter under external fields. This work may be useful for future magnetic information storage devices based on DW motion.

In the past few years, the manipulation of magnetic domain wall (DW) motion has attracted intensive attention for its fundamental interest and potential impacts in logic operations and data storage devices^1^,^2^,^3^,^4^,^5^,^6^,^7^,^8^,^9^,^10^,^11^,^12^,^13^,^14^,^15^,^16^,^17^. The DW motion can be controlled so far by static magnetic fields^1^,^2^,^3^,^4^, spin transfer torque^5^,^6^,^7^,^8^,^9^,^10^, microwaves or field pulses^11^,^12^, and spin waves (magnons)^13^,^14^,^15^,^16^,^17^,^18^. Recently, a lot of experiments have uncovered that the Dzyaloshinskii-Moriya interaction (DMI)^19^,^20^ plays a crucial role in stabilizing the chiral spin textures such as spin spirals^21^,^22^, homochiral DWs^23^,^24^,^25^ and skyrmions^26^,^27^. In addition, recent theoretical results predicted that the DMI has enormous potential to influence DW motion if it can be controllably manipulated^17^,^28^,^29^. The DMI is an antisymmetric exchange interaction which results from spin-orbit scattering of electrons in lattices or at the interface of noncentrosymmetric magnetic materials because their crystals are lacking structural inversion symmetry^23^,^30^. The DMI between two atomic spins ***S***_*i*_ and ***S***_*j*_ located on neighboring atomic sites *i* and *j* can be written as: 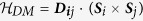, where ***D***_***i**j*_ is the DMI vector^30^,^31^,^32^,^33^. Therefore, the DMI prefers to make atomic spins on neighboring sites to be mutually perpendicular and leads to a chiral noncollinear spin structures when it competes with the exchange coupling. The direction of ***D***_***i**j*_ depends on the type of system considered. In this work we will focus on the so-called bulk DMI which was found in bulk noncentrosymmetric magnetic materials^34^,^35^. The vector ***D***_***i**j*_ is parallel to the unit vector ***u***_***i**j*_ joining the site of *i* and *j*, which is different from another so-called interface DMI which can be observed directly in ultrathin films by Brillouin light spectroscopy where ***D***_***i**j*_⊥***u***_***i**j*_^29^,^33^,^36^,^37^. The bulk DMI has micromagnetic energy density in continuous form *ε*_*DMI*_ = *D**m*** ⋅ (∇ × ***m***), where *D* is the DMI constant and ***m*** is the normalized magnetization vector ***m*** = ***M***/*M*_*s*_. Here ***M*** is a local magnetization vector with a saturation magnetization *M*_*s*_.

In this paper, we theoretically and numerically investigated the influence of the bulk DMI on field-driven DW motion in ferromagnetic nanowires with two magnetic anisotropy coefficients (biaxial wire), one for the easy-axis anisotropy along z-axis and another for hard-axis along y-axis as shown in Fig. 1(a). The cases with and without the hard-axis anisotropy are discussed in detail respectively. In the absence of the hard anisotropy, a new trial function was adopted and an exact solution was then obtained for the DW motion induced by an external field along the wire. The DW average velocity was found to be proportionally to the external fields as the DW width depends on the strength of the DMI constant *D*. Hence, a new strategy to speed up the DW motion in a uniaxial magnetic nanowire was suggested by increasing the DMI parameter which might be realized through some methods, for example, by interface engineering^30^,^32^. For the biaxial anisotropy case, an approximation method of generalized coordinates was applied^28^,^38^,^39^. It was uncovered that the breakdown field and velocity of the DW motion was strongly affected by the strength and sign of the DMI parameter under external fields. Furthermore, our theoretical results had been verified by solving the Landau-Lifshitz-Gilbert (LLG) equation numerically in a one-dimensional (1D) spin chain model. This discovery could be some help for future magnetic DW information storage devices.

## Model and Analytical Results

**Figure 1:**
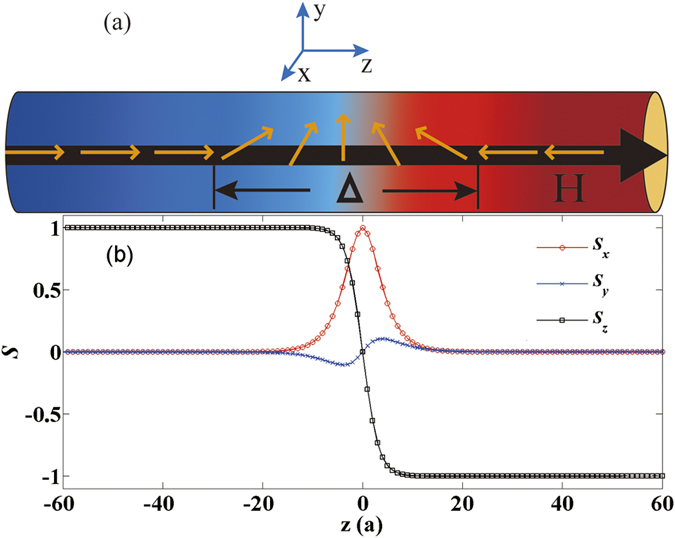
(**a**) Sketch of the nanowire with a head-to-head domain wall (DW) using yellow arrows. The blue and red regions denote domains on both sides of the wire. The color transition area denotes DW. The black arrow denotes the external magnetic field. (**b**) The snapshot of initial spin ***S*** components near the DW center for *α* = 0.1, *J* = 20, *K*_*z*_ = 1 and *D* = 1.

We considered a ferromagnetic nanowire which was modeled as a 1D classical biaxial spin chain along z direction as shown in Fig. 1(a). The continuous free energy density (per cross-sectional area) for the wire under an external field **H** is^17^,^28^


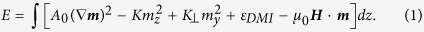


The first term of *E* in the integral is the exchange energy density with exchange coefficient *A*_0_ > 0. The second and third terms describe easy- and hard-axis anisotropy energy densities with coefficients *K*, *K*_⊥_ > 0, and the last term is the Zeeman energy density. *ε*_*DMI*_ is the bulk DMI term introduced previously.

The spatiotemporal dynamics of the normalized magnetization ***m*** is governed by the LLG equation^12^,^13^,


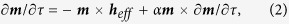


where *τ* = (*γM*_*s*_)^−1^*t* is the normalized time and *γ* is the gyromagnetic ratio. *α* is phenomenological Gilbert damping constant and ***h***_*eff*_ is the effective field given by the functional derivative of free energy density with respect to magnetization, 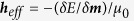, where *μ*_0_ is the vacuum permeability. The first term on the right hand side of the LLG equation describes a precessional motion of ***m*** and the second term describes the relaxation motion.

A usual spherical coordinates of polar angle *θ* and azimuthal angle *φ* relative to z axis was employed. Thus, ***m*** is expressed as ***m*** = (sin *θ* cos *φ*, sin *θ* sin *φ*, cos *θ*) and the effective field reads 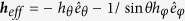, where


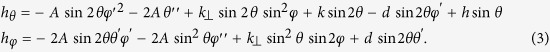


Here we have defined 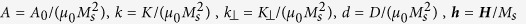
and chosen the external field along z axis (i.e.,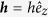). The symbols ′ and ′′ denote the partial and second partial derivative in z component. The dynamical equation (2) then takes the form


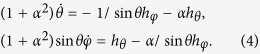


Initially, we considered the uniaxial anisotropy situation (i.e.,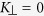) with a rotational symmetry around z axis. Without the external field and the DMI, the static DW profile follows the well-known Walker solution 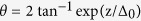, where 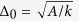 is the width of the DW^40^,^41^. When the external field and the DMI are taken into account, a new trial function is adopted as follow,


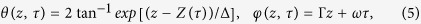


where Γ = *d*/2*A*. 
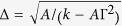
 describes the new DW width with the DMI^28^. *Z*(*τ*) is the DW center position defined as the location where the spin has zero z-component. One should note that *φ* is assumed to vary with time and space which is different from the usual Walker solution. Moreover, the DW profile Eq. (5) is only allowed if the DMI coefficient is smaller than the critical value 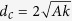 under the condition of spiral magnetization state being stable^28^.

By substituting the trial function Eq. (5) into Eq. (4) with *k*_⊥_ = 0, the solution can be found,


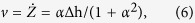



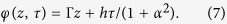


Here an exact solution of Eq. (4) is given only when *K*_⊥_ = 0. One should note that the exact solution with the DMI is different from the well-known steady solution, *v* = (*γα*Δ_0_)/(1 + *α*^2^)*H* and *φ*(*t*) =* φ*(0) +* *(*γH*)/(1* *+ *α*^2^)*t*, for a uniaxial anisotropy obtained by Slonczewski^41^. Firstly, the DW width is larger when the DMI is taken into account. As a result, the new DW velocity is larger by a factor of 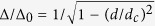 compared to the case without the DMI under the same field magnitude. To give a practical example, the parameters of FeGe is used and the DW velocity is increased by a factor of 2^17^. Thus, a new method to speed up the DW motion in a uniaxial magnetic nanowire can be proposed by increasing the DMI parameter. Secondly, the azimuthal angle *φ* with the DMI depends linearly on time and space, but *φ* without DMI is spatially constant (i.e., ∂*φ*/∂z =* *0) and increases linearly only with time. Finally, when we let *d* = 0 in Eqs (6) and (7) it returns to the form obtained by Slonczewski. That is to say, our results with the DMI have included the case without DMI.

Next, we considered the case of a ferromagnetic nanowire with biaxial anisotropy. The DW profiles are described by Eq. (4) and the DW width Δ is not a constant for the present case^17^, therefore our approach for the uniaxial anisotropy situation cannot be straightforwardly extended to the biaxial case. Nevertheless, if applied external field and hard-anisotropy coefficient is small we can consider the DW width Δ as invariant (i.e., 
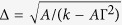
). Then we applied the method of generalized coordinates developed by Tretiakov *et al.* where two zero modes in the method correspond to the translation of the DW along the wire and its rotation around the wire axis of magnetization texture in 1D nanowires^28^,^38^,^39^.

For the 1D uniaxial spin chain with the DMI the free energy density takes the form 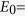 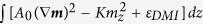. There exists a static head-to-head DW profile for the static LLG equation


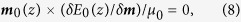


which can be expressed as^28^


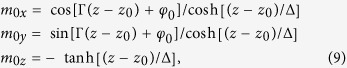


where *z*_0_ represents the DW center position and *φ*_0_ represents the tilt of the DW. The static profile corresponds to two zero modes of the system which are the most relevant modes if the system is perturbed. Then, the perturbed system can be represented in the form of the two zero modes with a small correction resulted from the perturbation. In our system, the perturbed terms are the external field and the hard-anisotropy term. Some details of the method and calculations were described in the supplementary material. Illustration and discussion of the results are as followed.

When the external field met *h* ≤ *h*_*c*_, we expected that the DW only moves along the wire and does not rotate around the axis of the wire, 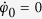, where


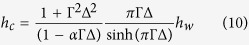


is called as breakdown field. *h*_*w*_ = *αk*_⊥_ is the well-known Walker breakdown field^40^,^41^. Thus, the DMI decreases the breakdown field, *h*_*c*_ < *h*_*w*_, because of 
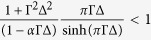
. The equations for the DW velocity is


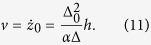


Thus, the DW velocity *v* increases linearly with *h* and the maximum velocity is


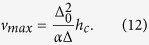


On the other hand, when *h* is larger than *h*_*c*_, the DW width Δ and its tilt angle *φ*_0_ is time-dependent (i.e.,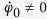), but the DW was found to make an oscillation motion by the following numerical study. The average velocity of the DW can be rewritten as


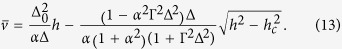


For the average velocity Eq. (13), firstly it returns to Eq. (6) if we consider the uniaxial anisotropy situation, *k*_⊥_ = 0 or *h*_*c*_ = 0. It is easily noticed that Eq. (11) and (13) equivalent at breakdown field, *h* = *h*_*c*_. So Eq. (13) is consistent with the previous results. When the external field *h* is above *h*_*c*_, the DW velocity decreases because the DW not only translates along the wire but also rotates around the wire axis which consumes a small percentage of energy from the external field.

## Numerical Results and Discussion

Until now, we had theoretically studied field-driven DW motion in a 1D biaxial ferromagnetic nanowire with the DMI and used some approximations to simplify the model. In general, a micromagnetic simulation is required to verify the validity of the findings under realistic situation^42^. Thus, Eq. (2) was solved numerically in a 1D nanowire here. In our simulations, the time, length, field amplitude and energy density are in the units of (*γM*_*s*_)^−1^, *a*, *M*_*s*_ and 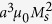, respectively. The wire length was chosen to be 10000 (from *n* = −5000 to *n* = 5000) with the unit length on a simple cubic lattice and the crystal constant is *a*. Initially, a static head-to-head DW was placed at the wire center (*n* = 0). Meanwhile, an absorbing boundary condition was adopted to avoid spin wave reflection at both ends by taking a large damping constant (*α* = 1) near the ends.

We now considered a classical Heisenberg biaxial chain along z direction with the DMI in an external field ***H***^43^,^44^


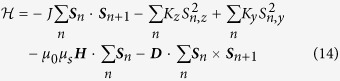


where ***S***_*n*_ is unit spin direction at the site *n*. The first term in Eq. (14) is ferromagnetic coupling between nearest neighboring lattice sites and *J* > 0 is ferromagnetic coupling constant. The second and third sum describe easy z-axis anisotropy and hard y-axis anisotropy with *K*_*z*_, *K*_*y*_ > 0. The fourth sum gives the Zeeman energy term and the last term describes the micromagnetic energy density produced by the DMI where ***D*** is the DMI vector.

It is firstly considered the uniaxial anisotropy case with parameters *a* = 0.1, *J* = 20, *K*_*z*_ = 1, *D* = 1 and *K*_*y*_ = 0. The snapshot of the initial spin profile near the DW center was shown in Fig. 1(b). It satisfies a static head-to-head DW profile with the DMI, 
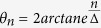
 and 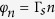, where the DW width 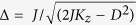 and Γ_*s*_ = *D*/*J*.

**Figure 2:**
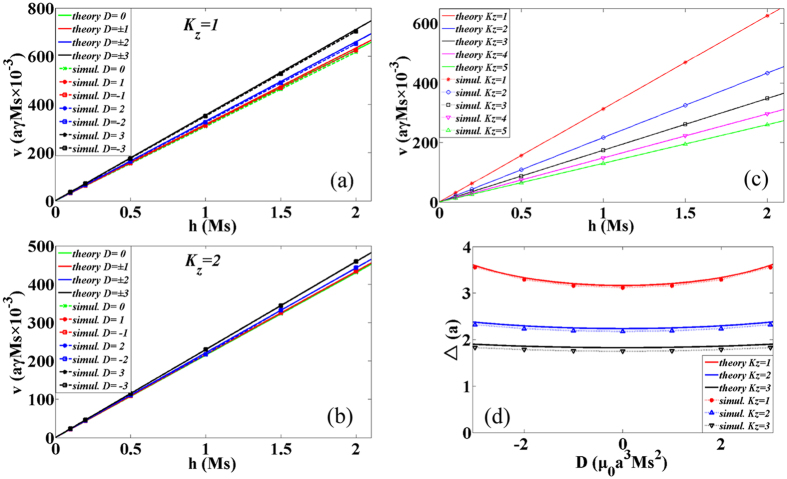
Field dependence of DW average velocity for different DMI constants with *α* = 0.1, *J* = 20, *K*_*z*_ = 1 in (**a**) and *K*_*z*_ = 2 in (**b**) in the absence of the hard-axis anisotropy coefficient. The solid lines are from solution of Eq. (6). (**c**) Field dependence of DW average velocity for different easy anisotropies. Also, the solid lines are from solution of Eq. (6). (**d**) The DMI constants dependence of DW width for different easy anisotropies. The solid curves denote the theoretical result 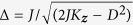.

We now studied the dynamics of the DW under a field ***H*** applied along the easy (z) axis which induces DW motion. In the following, the solid curves represented the analytical results and the scattered points were the numerical solutions. The average velocity of the DW as a function of field with different DMI constants and different easy anisotropy coefficients for *K*_*z*_ = 1 was shown in Fig. 2(a) and for *K*_*z*_ = 2 was shown in Fig. 2(b). As discussed in the previous results, the average DW velocity is linearly related to the external field with different DMI constants. The DMI increases the DW width as well as increases the velocity, thus the DW motion can be accelerated by increasing the strength of *D*. The solid lines shown in Fig. 2(a,b) were from our theoretical result Eq. (6). In Fig. 2(c), the field dependence of the DW average velocity was shown with different easy z-axis anisotropy *K*_*z*_. The theoretical predictions from Eq. (6) were shown by the solid lines. The average velocity is directly proportional to the external field and monotonically decreasing as *K*_*z*_ increases because the DW width gradually decreases. The relationship between the DW width and the DMI constants was also analyzed in Fig. 2(d) for several *K*_*z*_. The theoretical result 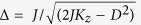 was plotted with solid curves.

**Figure 3:**
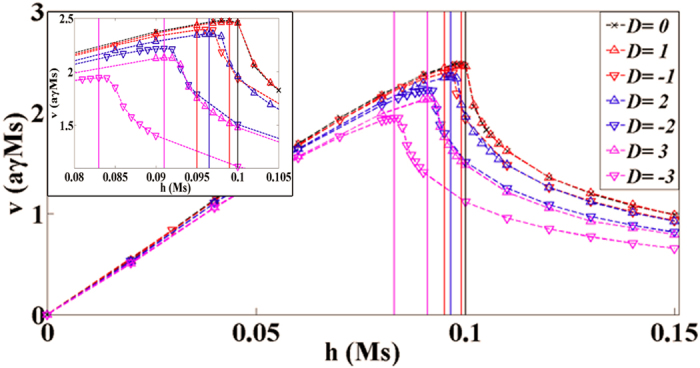
Field dependence of DW average velocity for different DMI constants with *a* = 0.1, *J* = 20 and *K*_*z*_ = 1 in the presence of the hard-axis anisotropy *K*_*y*_ = 1.The vertical solid lines denote breakdown fields. The dash curves are fitted curves. Inset: Enlarged figure near the breakdown fields.

Next, hard y-axis anisotropy *K*_*y*_ was taken into account. In Fig. 3, the field dependence of the average DW velocity with *K*_*y*_ = 1 was plotted with various values of *D*. The dash curves are fitted curves. The inset of Fig. 3 shows an enlarged view near the breakdown fields. In Fig. 3, breakdown fields for field-driven DW motion with the DMI were marked by vertical solid lines. When the external field is below the breakdown field, the velocity is approximately proportional to field strength as the slope gradually decreases approaching to the breakdown field. The velocity reaches a maximum *v*_*max*_ at the point that the external field is equal to the breakdown field. Then the DW velocity reduces gradually with increasing field. One should note that our findings about field-driven DW motion with the bulk DMI is very different from the previous work by Thiaville *et al.* as regard to the interface DMI^29^. They found that the Walker field is nearly linear increasing with positive *D*. Here in our study about the bulk DMI, the breakdown field is not only extremely relevant to the magnitude and sign of *D* but also smaller than the case without the DMI.

**Figure 4:**
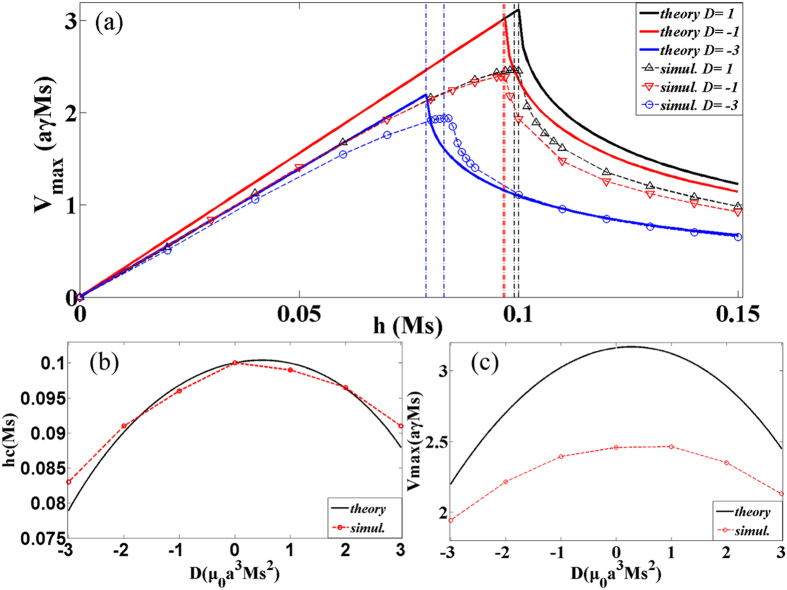
(**a**) Field dependence of DW average velocity for different DMI constants with *α* = 0.1, *J* = 20, *K*_*y*_ = 1 and *K*_*z*_ = 1. The solid curves denote the theoretical results from Eqs (11) and (13) and the scattered points are the numerical solutions. The dash curves are fitted curves. The vertical dash-dotted lines denote the positions of breakdown fields. (**b**) The DMI constants dependence of breakdown field for the same cases as those in (**a**). The solid curve is analytical result of Eq. (10). (**c**) The DMI constants dependence of maximum average velocity for the same cases as those in (**a**). The solid curve is analytical result of Eq. (12).

Lastly, we make a comparison between the theoretical and numerical results for biaxial anisotropy situation as shown in Fig. 4. The field dependence of the average DW velocity was plotted in Fig. 4(a). The solid curves denote the theoretical results from Eqs (11) and (13) and the scattered points are the numerical solutions. The vertical dash lines are positions of breakdown fields. The comparison about the breakdown field and the maximum average velocity was shown in Fig. 4(b,c). The solid curves in Fig. 4(b,c) are analytical results of Eqs (10) and (12). Based on a series of comparison and analysis, some interesting results were observed. Firstly, the breakdown field decreases with the increasing of the DMI strength *D* but no simple linear relationship exists between them. Moreover, the breakdown field with a negative *D* is smaller than a positive *D* in Fig. 4(b). Then, as *D* increases, *v*_*max*_ decrease rapidly and the maximum average velocity with a negative *D* is also smaller than one with a positive *D*. They are very different from the situation under the interface DMI^29^,^45^,^46^. The reason of some deviation between theory and simulation might be that the DW width with biaxial coefficient should be larger than that with easy-axis anisotropy coefficient. If the DW width was considered as a variable in field-driven DW motion under biaxial anisotropy situation with DMI, a modified *q–Φ* model by adding new conjugated collective variables (DW width and amplitude of the DMI deformation) has been reported earlier^47^.

## Conclusions

We had theoretically and numerically investigated the influence of the bulk DMI on field-driven DW motion in ferromagnetic nanowires. The uniaxial anisotropy and biaxial anisotropy cases were studied. Under the case with uniaxial anisotropy, an exact solution was found where the DW velocity is proportional to the external field and the DW width depends on the strength of the DMI constant. Thus, a new strategy to speed up the DW motion in a uniaxial magnetic nanowire by increasing the DMI parameter was proposed. For the biaxial anisotropy case, we employed an approximation method and found that the field-driven motion of a DW is strongly affected by the strength and sign of the DMI constant with decreasing the breakdown field and velocity. A micromagnetic simulation was also taken to verify the validity of the theoretical findings under realistic situation. Our results may be useful for ultrahigh-density magnetic storage devices based on DW motion in practical materials with the DMI.

## Method

The analysis conducted in this work was based on a combination of theoretical analytical derivations and micromagnetic simulations. Our micromagnetic simulation code is written based on finite difference method. We use Runge-Kutta method to solve the LLG equations. In our simulations, the time, length, field amplitude and energy density are in the units of (*γM*_*s*_)^−1^, *a*, *M*_*s*_ and 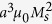, respectively. The wire length was chosen to be 10000 (from *n* =−5000 to *n* = 5000) with the unit length on a simple cubic lattice and the crystal constant is *a*. Initially, a static head-to-head DW was placed at the wire center (*n* = 0). An absorbing boundary condition was adopted to avoid spin wave reflection at both ends by taking a large damping constant (*α* = 1) near the ends.

## Additional Information

**How to cite this article**: Zhuo, F. and Sun, Z. Z. Field-driven Domain Wall Motion in Ferromagnetic Nanowires with Bulk Dzyaloshinskii-Moriya Interaction. *Sci. Rep.* **6**, 25122; doi: 10.1038/srep25122 (2016).

## Supplementary Material

Supplementary Information
