# Supplementary material for: Field-driven Domain Wall Motion in Ferromagnetic Nanowires with Bulk Dzyaloshinskii-Moriya Interaction
Source: Sci Rep. 2016 Apr 27;6:25122. doi: 10.1038/srep25122 (PMC4846831; doi:10.1038/srep25122)
Supplement: Supplementary Information [file srep25122-s1.pdf]

Supplemental Material for "Field-driven Domain Wall Motion in Ferromagnetic Nanowires with Bulk Dzyaloshinskii-Moriya Interaction"

Fengjun Zhuo[1,2] and Z. Z. Sun[1,*]

[1] College of Physics, Optoelectronics and Energy & Jiangsu Key Laboratory of Thin Films, Soochow University, Suzhou, Jiangsu 215006, China

[2] Jiangsu Key Laboratory for Carbon-Based Functional Materials & Devices, Institute of Functional Nano & Soft Materials (FUNSOM), Soochow University, Suzhou, Jiangsu 215123, China

*phzzsun@suda.edu.cn

We applied the method of generalized coordinates[1-3] here to study the influence of the DMI on field-driven DW motion in ferromagnetic nanowires with biaxial anisotropy. When the applied external field and hard-anisotropy coefficient were taken into account, the total free energy density takes the form

$$E = \int \left[A_0 (\nabla \boldsymbol{m})^2 - K m_z^2 + K_\perp m_y^2 + \varepsilon_{DMI} - \mu_0 \boldsymbol{H} \cdot \boldsymbol{m} \right] dz. \quad \text{(S1)}$$

We divided the total free energy density into two parts, $E = E_0 + e$, where $E_0 = \int [A_0 (\nabla \boldsymbol{m})^2 - K m_z^2 + \varepsilon_{DMI}] dz$ is the 1D uniaxial spin chain with the DMI the free energy density and $e(z) = \int \left[K_\perp m_y^2 - \mu_0 \boldsymbol{H} \cdot \boldsymbol{m} \right] dz$ is the perturbed terms. e is expected much smaller than E_0 so that there is a small correction $\boldsymbol{h}_1$ to the static LLG equation

$$\boldsymbol{m}_0(z) \times (\delta E_0(z) / \delta \boldsymbol{m}) / \mu_0 = 0. \quad \text{(S2)}$$

Then a perturbed LLG equation is given

$$\dot{\boldsymbol{m}} = -\boldsymbol{m} \times (\delta E_0(z)/\delta \boldsymbol{m})/\mu_0 \quad + \boldsymbol{h}_1, \tag{S3}$$

where

$$\boldsymbol{h}_1 = \alpha \boldsymbol{m} \times \dot{\boldsymbol{m}} - \boldsymbol{m} \times (\delta e(z)/\delta \boldsymbol{m})/\mu_0. \tag{S4}$$

We now assumed $\boldsymbol{m}$ has the form

$$\boldsymbol{m}(z,\tau) = \boldsymbol{m}_0\big(z, z_0(\tau), \varphi_0(\tau)\big) + \boldsymbol{m}_1(z,\tau), \tag{S5}$$

where z_0, φ_0 and $\boldsymbol{m}_1$ slowly vary in time, and $\boldsymbol{m}_1$ is much smaller than $\boldsymbol{m}_0$ at all times. Using the method of generalized coordinates and through a straightforward calculation, we found

$$(1 - \alpha\Gamma\Delta)\dot{z}_0 = -\alpha\Delta\dot{\varphi}_0 + \pi k_\perp \Gamma \Delta^2 \sin(2\varphi_0)/\sinh(\pi\Gamma\Delta), \tag{S6}$$

$$(1 + \alpha\Gamma\Delta)\dot{\varphi}_0 = \alpha\Delta\dot{z}_0/\Delta_0^2 - h. \tag{S7}$$

When the external field is small, the DW is expected only moves along the wire and does not rotate around the axis of the wire. Hence, $\dot{\varphi}_0 = 0$ holds in Eqs. (S6) and (S7). Then we found

$$v = \dot{z}_0 = \frac{\Delta_0^2}{\alpha\Delta} h, \tag{S8}$$

$$h = \frac{1+\Gamma^2\Delta^2}{(1-\alpha\Gamma\Delta)} \frac{\pi\Gamma\Delta}{\sinh(\pi\Gamma\Delta)} h_w \sin(2\varphi_0), \tag{S9}$$

where $h_w = \alpha k_\perp$ is the well-known Walker breakdown field[4]. The first equation gives the DW propagation velocity and the second one gives its tilt angle φ_0. The formulas only hold when $|\sin(2\varphi_0)| \leq 1$. That is to say, the external field must meet $h \leq h_c$ where

$$h_c = \frac{1+\Gamma^2\Delta^2}{(1-\alpha\Gamma\Delta)} \frac{\pi\Gamma\Delta}{\sinh(\pi\Gamma\Delta)} h_w. \tag{S10}$$

h_c is referred to as the breakdown field. Therefore, when the external field h is less than h_c, the DW velocity v increases linearly with h and the maximum velocity is

$$v_{max} = \frac{\Delta_0^2}{\alpha\Delta} h_c. \tag{S11}$$

When h is larger than h_c, the DW width Δ and its tilt angle φ_0 is time-dependent (i.e., $\dot{\varphi_0} \neq 0$). The average velocity of the DW can be calculated as follows. Using the definition of h_c, Eq. (S10), Eqs. (S8) and (S9) can be rewritten as

$$\dot{\varphi_0} = -\frac{1-\alpha\Gamma\Delta}{1+\alpha^2}[h - h_c \sin(2\varphi_0)], \tag{S12}$$

$$\dot{z}_0 = \frac{\alpha\Delta}{1+\alpha^2} h + \frac{(1-\alpha^2\Gamma^2\Delta^2)\Delta}{\alpha(1+\alpha^2)(1+\Gamma^2\Delta^2)} h_c \sin(2\varphi_0). \tag{S13}$$

Eq. (S12) can be integrated over a period T of DW rotation

$$\int_0^{2\pi} \frac{d\varphi_0}{h/h_c - \sin(2\varphi_0)} = -\frac{1-\alpha\Gamma\Delta}{1+\alpha^2} h_c T, \tag{S14}$$

and we also have

$$h_c \int_0^T \sin(2\varphi_0)\, d\tau = T\left(h - \sqrt{h^2 - h_c^2}\right). \tag{S15}$$

Then Eq. (S13) can be integrated over a period T and use Eqs. (S14) and (S15) to obtain the average velocity which defined as $\bar{v} = \int_0^T \dot{z}_0 d\tau / T$,

$$\bar{v} = \frac{\Delta_0^2}{\alpha\Delta} h - \frac{(1-\alpha^2\Gamma^2\Delta^2)\Delta}{\alpha(1+\alpha^2)(1+\Gamma^2\Delta^2)} \sqrt{h^2 - h_c^2}. \tag{S16}$$

References

1. Tretiakov O.A. & Abanov, Ar. Current driven magnetization dynamics in ferromagnetic nanowires with Dzyaloshinskii. *Phys. Rev. Lett.* 105, 157201 (2010).

2. Tretiakov, O. A. Clarke, D. Chern, G. W. Bazaliy, Ya. B. & Tchernyshyov, O. Dynamics of Domain Walls in Magnetic Nanostrips. *Phys. Rev. Lett.* 100, 127204 (2008).

3. Clarke, D. J. Tretiakov, O. A. Chern, G. W. Bazaliy, Ya. B. & Tchernyshyov, O. Dynamics of a vortex domain wall in a magnetic nanostrip: an application of the collective coordinate approach. *Phys. Rev. B.* 78, 134412 (2008).

4. Schryer, N. L. & Walker, L. R. The motion of 180° domain walls in uniform dc magnetic fields. *J. Appl. Phys.* 45, 5406 (1974).
